# Sleep spindles in people with schizophrenia, schizoaffective disorders or bipolar disorders: a pilot study in a general population-based cohort

**DOI:** 10.1186/s12888-022-04423-y

**Published:** 2022-12-03

**Authors:** Jean-Marie Petit, Marie-Pierre F. Strippoli, Aurélie Stephan, Serateh Ranjbar, José Haba-Rubio, Geoffroy Solelhac, Raphaël Heinzer, Martin Preisig, Francesca Siclari, Kim Q. Do

**Affiliations:** 1 Center for Psychiatric Neuroscience (CNP), CHUV, Department of Psychiatry, Lausanne University Hospital and University of Lausanne, Route de Cery 11c, CH-1008 Prilly, Switzerland; 2 Center for Psychiatric Epidemiology and Psychopathology (CEPP), Department of Psychiatry, Lausanne University Hospital and University of Lausanne, Prilly, Switzerland; 3 Center for Sleep Research and Investigation (CIRS), Lausanne University Hospital and University of Lausanne, Lausanne, Switzerland

**Keywords:** Psychosis, EEG, Manic symptoms, Endophenotype, Non-REM stage 2, Sigma frequency band

## Abstract

**Background:**

Sleep spindles have been involved in sleep stabilization and sleep-related memory mechanisms and their deficit emerged as possible biomarker in schizophrenia. However, whether this sleep phenotype is also present in other disorders that share psychotic symptoms remains unclear. To address this gap, we assessed sleep spindles in participants of a prospective population-based cohort who underwent psychiatric assessment (CoLaus|PsyCoLaus) and polysomnographic recording (HypnoLaus).

**Methods:**

Sleep was recorded using ambulatory polysomnography in participants (*N* = 1037) to the PsyCoLaus study. Sleep spindle parameters were measured in people with a lifelong diagnosis of schizophrenia (SZ), schizoaffective depressive (SAD), schizoaffective manic (SAM), bipolar disorder type I (BP-I) and type II (BP-II). The associations between lifetime diagnostic status (independent variables, SZ, SAD, SAM, BPD-I, BPD-II, controls) and spindle parameters (dependent variables) including density, duration, frequency and maximum amplitude, for all (slow and fast), slow- and fast-spindle were assessed using linear mixed models. Pairwise comparisons of the different spindle parameters between the SZ group and each of the other psychiatric groups was performed using a contrast testing framework from our multiple linear mixed models.

**Results:**

Our results showed a deficit in the density and duration of sleep spindles in people with SZ. They also indicated that participants with a diagnosis of SAD, SAM, BP-I and BP-II exhibited different sleep spindle phenotypes. Interestingly, spindle densities and frequencies were different in people with a history of manic symptoms (SAM, BP-I, and BP-II) from those without (SZ, SAD).

**Conclusions:**

Although carried out on a very small number of participants due to the low prevalence of these disorders in general population, this pilot study brought new elements that argued in favor of a deficit of sleep spindles density and duration in people with schizophrenia. In addition, while we could expect a gradual change in intensity of the same sleep spindle parameters through psychotic diagnoses, our results seem to indicate a more complex situation in which the frequency of sleep spindles might be more impacted by diagnoses including a history of mania or hypomania. Further studies with a larger number of participants are required to confirm these effects.

**Supplementary Information:**

The online version contains supplementary material available at 10.1186/s12888-022-04423-y.

## Background

Sleep and psychiatric disorders are often comorbid and sleep symptoms are even part of the diagnostic criteria for manic and depressive episodes. Macroarchitecture impairments such as total sleep time reduction, increase in sleep onset latency, changes in rapid eye movement (REM)-sleep, have been observed in patients with various psychiatric disorders including major depressive disorder, autism, bipolar disorders, and schizophrenia spectrum disorders [1]. However, alterations of sleep microarchitecture (i.e sleep stage-dependent oscillatory rhythms) reflecting particular brain networks alterations, appear to be more restricted to specific disorders and could be used as biomarkers, as suggested for sleep spindles in schizophrenia [2]. Sleep spindles, known as brief events detected in sleep electroencephalographic (EEG) recordings, constitute, together with K-complexes, the hallmarks of the N2 stage of non-REM (NREM) sleep. They correspond to EEG oscillations in the sigma range (10-16 Hz) characterized by a gradual increase in amplitude (“waxing “) followed by their decrease (“waning”). Furthermore, based on their main frequency, we can distinguish slow spindles (< 14 Hz) and fast spindles (≥14 Hz) which exhibit a specific brain topography with slow spindles predominating over frontal and central regions whereas fast spindles predominating over centro-parietal and occipital areas [3]. The origin of sleep spindles has been extensively described [4–6] and involves local thalamo-cortical loops including GABAergic neurons of the thalamic reticular nucleus (TRN), thalamo-cortical glutamatergic neurons of the thalamus (TC) and cortical glutamatergic neurons (mainly from cortical layer VI). In these networks, the capacity to generate bursts of activity at the origin of cortical spindles is thought to be mainly due to the low-threshold Ca^2+^ channels, more specifically Ca_V_3.3 channels [7], and to the Ca^2+^-dependent K^+^ channels of SK2-type [8], both present on the dendrites of GABAergic cells of the TRN.

Sleep spindles gained attention mainly due to their involvement in memory-related sleep mechanisms and their association with cognitive skills in humans [9–12]. Electrophysiological experiments in rodents and humans indicated that spindles, up-states of cortical slow oscillations (< 1 Hz) and hippocampal sharp wave-ripples (brief 80–200 Hz bursts at CA1 location) displayed a time-locked occurrence [13–16]. Based on these results, sleep spindles are thought to mediate the hippocampal-neocortical dialogue involved in the engram formation [15, 17]. As people with schizophrenia spectrum disorders frequently experience sleep problems and cognitive impairment, many investigations focused on the association between these disorders and sleep spindle parameters. When computerized methods were used to quantify sleep spindles, the vast majority of studies found spindle density to decrease in people with schizophrenia when compared to healthy controls [18–24]. Similar decrease has also been reported for spindle amplitude and spindle duration [23, 25], which have also been shown to be associated with cognitive dysfunction [19, 26, 27]. However, it remains unclear whether sleep spindle deficiency is a specific marker for schizophrenia or whether it also occurs more broadly in people with schizophrenia spectrum disorder such as schizoaffective disorders. Although existence of schizoaffective disorders as real and operative diagnostic entity remains controversial [28–30], their diagnosis corresponds to patients who display a mixture of psychotic features related to schizophrenia and of mood symptoms related to bipolar disorders. Therefore, this diagnosis can include a diversity of symptoms fluctuating between schizophrenia (SZ) and mood disorders. According to the DSM-5 [31], schizoaffective disorders can be sub-divided into a depressive (SAD) and a bipolar (or manic) subtype (SAM) depending on the lifetime occurrence of manic and depressive mood symptoms.

Several studies suggest that schizophrenia and bipolar disorders partially share etiological factors at genetic and biological levels. Therefore, investigating sleep spindle in bipolar disorders might help us to know whether patients with bipolar disorders reveal alterations of sleep spindle characteristics similar to those observed in those with schizophrenia. Only two studies with conflicting results have so far compared sleep spindles characteristics in bipolar patients and controls. One of them did not find a significant differences in any spindle parameters between patients with bipolar disorders and controls [32] whereas the other one revealed a decrease in fast-spindle density and mean intra-spindle frequency in bipolar outpatients [33]. In addition, these two studies did not distinguish between the bipolar I (BP-I) and bipolar II (BP-II) subtypes.

To our knowledge, no community study has yet assessed the specific associations of schizophrenia spectrum and bipolar disorder with sleep spindles. To bridge this gap, we assessed sleep spindles in participants of a population-based cohort who underwent both a psychiatric evaluation and an ambulatory polysomnographic recording (PSG). We compared the various parameters of sleep spindles across people with lifetime diagnoses of SZ, SAD, SAM, BP-I, BP-II and controls.

## Methods

### Participants

CoLaus|PsyCoLaus is a prospective cohort study designed to investigate mental disorders and their association with cardiovascular risk factors in the general population [34, 35]. The initial cohort of 6734 participants was randomly selected from the residents of the city of Lausanne (Switzerland) aged between 35 and 75 years in 2003 according to the civil registry. After the baseline investigation involving physical and psychiatric evaluations, participants were followed-up after approximately 5 (follow-up 1, FU1), 9 (follow-up 2, FU2) and 13 years (follow-up 3, FU3).

Between 2009 and 2013 (FU1), 2162 participants among the participants to CoLaus|PsyCoLaus, also underwent a full-night polysomonographic recording at home to assess objective sleep variables (HypnoLaus study) [36]. In order to determine the diagnosis at the moment of PSG, psychiatric information was used from the subsequent psychiatric evaluation (FU1 or FU2). Participants with incomplete sleep investigation (*n* = 6) or subsequent psychiatric information (*n* = 232) and those who met lifetime criteria for major depressive disorder (*n* = 887) were excluded from the present analysis leaving a final sample of 1037 participants (41.7% women, mean (s.d.) age at sleep evaluation 59.8 (11.3) years). Among this 1037 participants, the number of people in the different groups were: SZ *n* = 7, SAD *n* = 12, SAM *n* = 4, BP-I *n* = 17, BP-II *n* = 9 and CTL *n* = 988.

### Psychiatric evaluation

Participants were interviewed by psychologists, who were trained over a one- to two-month period. In order to provide ongoing supervision throughout the study, an experienced senior psychologist reviewed each interview. Diagnostic information on mental disorders at baseline and follow-up was collected using the semi-structured Diagnostic Interview for Genetic Studies (DIGS) [37]. The French version of the DIGS revealed excellent inter-rater and fair to good test–retest reliability for psychotic and mood disorders [38, 39]. At follow-up, a shortened version of the DIGS was used, which assessed the occurrence of psychopathology since the last assessment. Cumulative lifetime diagnoses at the time point of the HypnoLaus visit were assigned according to the combination of information from available subsequent diagnostic interviews with a participant. Non-mood diagnoses were defined according to the DSM-IV, whereas diagnoses of mood disorders were defined according to the DSM-5.

### Sleep recordings and sleep parameters analysis

A full night ambulatory PSG was performed using a portable sleep/wake recording system (EMBLA Titanium®, Embla systems, Inc., Broomfield, CO, USA). Electroencephalogram (EEG) from frontal (F3, F4), central (C3, C4), and occipital (O1, O2) areas, right and left electro-oculogram (EOG) and submental electromyogram (EMG) were recorded for sleep parameter analysis (see Supplement 1 for more details). From these recordings, usual sleep variables were calculated (see Supplement 2). The total sleep time (TST) and the percentage (relative to TST) of Non-REM stage 2 (N2%) as well as the apnea/hypopnea index (AHI) scored according to the American Association of Sleep Medicine rules (2013) were included as independent variables in our linear mixed models for sleep spindles analysis.

### Sleep spindles analysis

Sleep spindles during N2 were detected automatically from the EEG signal using an algorithm adapted from a previous study [25]. The EEG signal was average-referenced, downsampled to 128 Hz and bandpass-filtered between 11 and 16 Hz (∼3 dB at 10 and 17 Hz). A b-spline wavelet was applied to the filtered data of each channel separately. The power of the signal was then measured by squaring and smoothing the data with a 100 ms sliding window. We first defined spindles as sections with a power above two times and under six times the mean of the subject’s detection in each channel power (subject-based bound). In a second step, since some events that were not identified as spindles upon visual inspection remained, we selected spindles with duration of 0.5 to 3 seconds and amplitude less than or equal to the 3rd quartile + 1.5 times the interquartile range of the distribution of amplitude values measured in all HypnoLaus participants (population-based upper bound).

Intra-spindle frequency (Hz) (named “spindle frequency” in following parts of the manuscript) and density (i.e. number of spindles/min) were also calculated. According to their mean frequency, spindles in the slow (mean frequency < 14 Hz) and fast (mean frequency ≥ 14 Hz) ranges were also separately analyzed from the EEG signal recorded at F3, F4, C3 and C4 locations. Such a threshold was already used by Ferrarelli et al. for spindles analysis on people with schizophrenia. Data from O1 and O2 were omitted from the analysis to avoid artefacts due to contamination by bursts of alpha activity over occipital cortices that may mimic spindles. We used a mixed model including electrodes location in the fixed part of the model accounting for the area specific effect in the brain.

### Other assessments

Medication collected during CoLaus|PsyCoLaus investigations was coded according to the World Health Organization ATC classification (http://www.whocc.no/atcddd). We considered hypnotics (ATC code: N05CF), benzodiazepines or derivates (N05BA, N05CD, N03AE), antidepressants (N06A) and neuroleptics (N05A) as the drug categories with a potential effect on sleep. Since nicotine may increase spindles [40], we also considered whether participants were smokers at the time of the sleep recordings. In addition, current smoking status and body mass index (BMI) (i.e. self-reported weight (kg) divided par the square of height (m)) were collected at the time of the sleep recordings.

### Statistical analysis

Univariate between-group analyses were performed using chi-square tests or ANOVA/Wilcoxon tests as appropriate. The mean (standard deviation) and median (interquartile range) of spindle density, duration, frequency, maximum amplitude as well as the Spearman’s rho correlation coefficients between electrodes location were provided. The associations between lifetime diagnostic status (independent variables, SZ, SAD, SAM, BPD-I, BPD-II, controls) and spindle parameters (dependent variables) including density, duration, frequency and maximum amplitude, for all (slow and fast), slow- and fast-spindle were assessed using linear mixed models. These models were serially adjusted by adding the confounding variables in our models and a random intercept to account for the intra-class correlation between the repeated observations of each participant: 1) Model 1 was adjusted for age, sex, BMI, current smoking status and electrode location; 2) Model 2 was additionally adjusted for sleep variables, (TST, N2%, AHI) and medication use (hypnotics, antidepressants, antipsychotics and neuroleptics). Since residuals obtained from the entire sample (*n* = 1037) did not follow normal distribution, density (fast) and duration (all, slow) were log-transformed and duration (fast) and maximum amplitude (all, slow, fast) were transformed using Box-Cox transformation to render the data more normal distribution like. To test whether participants with SAD, SAM, BP-I or BP-II differed from the participants with SZ, we used the contrast testing framework on the fitted models (see Supplement 3 for details). In addition, the adjusted *p*-values using stepwise Bonferroni-Holm were reported to account for multiple testing (see Supplement 3). Statistical analyses were conducted using Prism 9.1 for Windows (GraphPad Software Inc., San Diego, CA, USA) and the Statistical Analysis System (SAS Institute Inc., Cary, NC, USA) version 9.4 for Windows.

## Results

### Characteristics of participants

Table 1 presents the participants’ characteristics for the whole sample as well as by lifetime diagnostic groupings. The AHI scores, the current smoking status and the presence of medication differed between diagnostic groups.

**Table 1:** Characteristics of participants by lifetime diagnosis

		Lifetime diagnosis							
	ALL	SZ	SAD	SAM	BP-I	BP-II	CTL	χ^**2**^/F	***p-value***
N	1037	7	12	4	17	9	988		
**Socio-demographic factors**									
Age (yrs), mean (sd)	59.8 (11.3)	63.2 (12.6)	58.2 (12.4)	54.2 (6.7)	57.0 (11.0)	57.1 (9.9)	59.9 (11.3)	F_5_ = 0.7	*0.624*
Males, %	58.3	71.4	41.7	100.0	70.6	55.6	58.1	χ^2^_5_ = 5.8	*0.324*
**Somatic characteristics**									
BMI (kg/m^2^), mean (sd)	25.7 (4.0)	26.1 (7.5)	25.1 (4.3)	28.4 (3.6)	26.4 (4.2)	24.7 (4.7)	25.6 (4.0)	F_5_ = 0.7	*0.642*
**Sleep characteristics**									
TST (min), mean (sd)	394.4 (70.1)	373.1 (127.9)	386.4 (78.6)	328.4 (46.9)	398.2 (97.5)	404.1 (72.1)	394.8 (69.0)	F_5_ = 0.9	*0.468*
N2 (%), mean (sd)	46.7 (10.6)	45.4 (12.9)	41.6 (9.5)	43.1 (12.1)	44.6 (12.2)	45.6 (9.3)	46.9 (10.5)	F_5_ = 0.9	*0.508*
AHI, mean (sd)	16.7 (16.9)	36.8 (47.4)	8.8 (6.7)	8.6 (3.7)	14.4 (8.9)	15.0 (12.0)	16.8 (16.7)	F_5_ = 2.8	***0.016***
**Behavioral characteristics**									
Current smokers, %	15.6	14.3	33.3	50.0	47.1	11.1	14.8	χ^2^_5_ = 19.9	***0.001***
**Medication**									
Any medication^a^, %	6.1	42.9	25.0	25.0	35.3	0.0	5.1	χ^2^_5_ = 5.8	***< 0.001***

*SZ* Schizophrenia, *SAD* Schizoaffective disorder - depressive type, *SAM* Schizoaffective disorder - bipolar type, *BP-I* Bipolar type I disorder, *BP-II* Bipolar type II disorder, *CTL* Comparison participants, *yrs* years, *min* minutes, *BMI* Body mass index, *sd* standard deviation, *TST* Total sleep time, *N2*% of NREM-stage 2, *AHI* Apnea hypopnea index

^a^Medication including hypnotics (ATC code: N05CF), benzodiazepines or derivates (N05BA, N05CD, N03AE), antidepressants (N06A) and neuroleptics (N05A)

Measures given the sleep macroarchitecture are summarized in Supplementary Table 1 (see supplement 2 for terms definition). Remarkably, no significant difference between groups was observed excepted for the TRT that corresponds to the time from the moment the lights have been switched off and switched back on (Kruskall-Wallis test, F_5,1031_ = 13.0; *p* = 0.023).

### Sleep spindle analysis

**Fig. 1:**
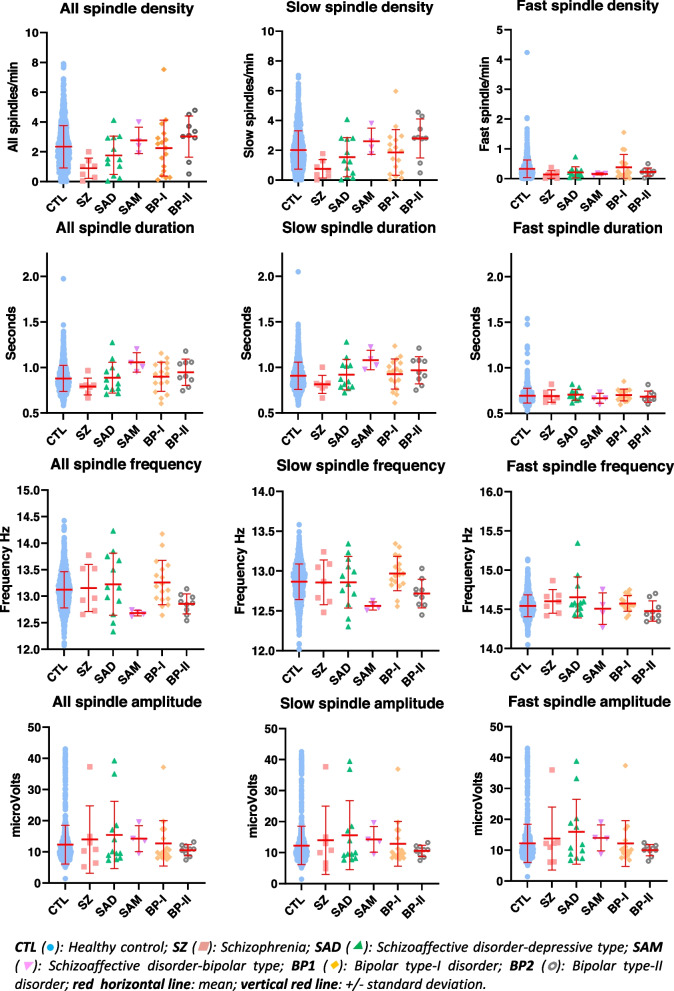
Distribution of the different sleep spindle parameter values in each diagnostic group measured at F4

The distribution of different sleep spindle features in the different lifetime diagnostic groups measured at F4 location that is representative of other electrodes which displayed a good correlation (see Supplementary Table 4), is presented in Fig. 1. Mean and median values of spindle features measured at each electrode location in participants with different disorders are presented in Supplementary tables 2 and 3 in the Additional file 1.

People with a life time diagnosis of SZ displayed a marked decrease in all sleep spindle density (50–66% depending on the electrode location; Fig. 1 and supplementary tables 2 and 3 in the Additional file 1). The associations between lifetime diagnosis of SZ, SAD, SAM, BP-I and BP-II and spindle parameters were presented in Table 2. After adjustment for age, sex, BMI, smoking status, electrode location, TST, N2%, AHI and medication, spindle density and duration for all spindles were significantly decreased in SZ group compared to the comparison probands. This effect is mainly supported by the decrease in slow spindle density and duration. While the density of fast spindle displayed only a tendency to decrease, their duration exhibited a slight but significant increase. No difference between the SZ and the comparison groups was observed for frequency and maximal amplitude whatever the type of spindles.

**Table 2:** Associations between sleep spindles parameters and lifetime diagnosis of psychiatric disorders (*n* = 1037)

	Lifetime diagnosis									
	SZ		SAD		SAM		BP-I		BP-II	
	β^**a**^ (95CI)	β^**b**^ (95CI)	β^**a**^ (95CI)	β^**b**^ (95CI)	β^**a**^ (95CI)	β^**b**^ (95CI)	β^**a**^ (95CI)	β^**b**^ (95CI)	β^**a**^ (95CI)	β^**b**^ (95CI)
**Density**										
all	− 1.18*** (− 1.64,-0.72)	−1.08*** (− 1.58,-0.57)	− 0.64° (− 1.36,0.08)	−0.62° (− 1.35,0.11)	0.28 (− 0.11,0.68)	0.23 (− 0.10,0.57)	0.18 (− 0.59,0.94)	0.17 (− 0.59,0.93)	0.20 (− 0.46,0.86)	0.21 (− 0.40,0.83)
slow	− 0.90*** (− 1.22,-0.58)	− 0.83*** (− 1.19,-0.47)	−0.48 (− 1.11,0.14)	−0.48 (− 1.11,0.15)	0.61** (0.19,1.02)	0.56** (0.18,0.93)	− 0.07 (− 0.53,0.38)	− 0.09 (− 0.54,0.36)	0.40 (− 0.18,0.97)	0.40 (− 0.15,0.96)
fast (log)	− 0.92* (− 1.82,-0.03)	−0.84° (− 1.74,0.05)	−0.47 (− 1.12,0.18)	−0.40(− 1.09,0.28)	− 0.39*** (− 0.58,-0.20)	−0.34*** (− 0.51,-0.17)	0.14 (− 0.29,0.58)	0.18 (− 0.29,0.64)	− 0.22(− 0.60,0.16)	− 0.22 (− 0.59,0.16)
**Duration**										
all (log)	−0.10** (− 0.18,-0.03)	−0.10** (− 0.17,-0.04)	0.01 (− 0.07,0.09)	0.02 (− 0.06,0.09)	0.13*** (0.07,0.19)	0.13*** (0.08,0.19)	− 0.01(− 0.07,0.05)	−0.01 (− 0.07,0.05)	0.03 (− 0.04,0.09)	0.03 (− 0.04,0.09)
slow (log)	−0.11** (− 0.20,-0.03)	−0.11** (− 0.18,-0.04)	0.01 (− 0.08,0.11)	0.02 (− 0.07,0.11)	0.12** (0.05,0.19)	0.12*** (0.06,0.18)	− 0.01 (− 0.07,0.06)	−0.01 (− 0.07,0.06)	0.02 (− 0.05,0.10)	0.03 (− 0.04,0.09)
fast (exp-2.5)	0.39° (− 0.01,0.78)	0.38* (0.01,0.75)	−0.15 (− 0.34,0.04)	−0.18* (− 0.36,-0.01)	0.03 (− 0.28,0.34)	0.01 (− 0.28,0.30)	− 0.07 (− 0.26,0.13)	−0.06 (− 0.27,0.14)	0.13(− 0.09,0.35)	0.12 (− 0.08,0.33)
**Frequency**										
all	0.05 (− 0.19,0.28)	0.05 (− 0.20,0.30)	0.15 (− 0.14,0.44)	0.18 (− 0.11,0.46)	− 0.39*** (− 0.53,-0.25)	−0.36*** (− 0.51,-0.22)	0.16* (0.02,0.30)	0.18* (0.02,0.33)	− 0.23*** (− 0.34,-0.13)	− 0.23*** (− 0.34,-0.13)
slow	0.00 (− 0.09,0.09)	0.01 (− 0.10,0.11)	0.04 (− 0.11,0.19)	0.05 (− 0.10,0.20)	−0.19*** (− 0.28,-0.10)	− 0.17*** (− 0.25,-0.09)	0.08* (0.01,0.14)	0.09* (0.02,0.16)	− 0.13*** (− 0.21,-0.06)	− 0.14***(− 0.20,-0.07)
fast	0.08 (− 0.03,0.19)	0.06 (− 0.04,0.17)	0.09°(0.00,0.19)	0.09°(0.00,0.19)	− 0.11*** (− 0.17,-0.05)	− 0.11*** (− 0.17,-0.04)	0.04 (− 0.03,0.10)	0.03 (− 0.03,0.10)	− 0.03 (− 0.11,0.05)	−0.03(− 0.11,0.05)
**Amplitude**										
all (exp-0.5)	0.00 (− 0.06,0.07)	0.01 (− 0.05,0.07)	− 0.01 (− 0.05,0.03)	−0.01 (− 0.05,0.03)	−0.04* (− 0.08,0.00)	−0.04° (− 0.09,0.00)	0.01 (− 0.02,0.03)	0.01 (− 0.02,0.03)	0.02° (0.00,0.03)	0.02°(0.00,0.03)
slow (exp-0.5)	0.00 (− 0.06,0.07)	0.01 (− 0.06,0.08)	−0.01 (− 0.05,0.03)	−0.01 (− 0.05,0.02)	−0.04* (− 0.09,0.00)	−0.04° (− 0.09,0.00)	0.01 (− 0.02,0.03)	0.01 (− 0.02,0.03)	0.02° (0.00,0.03)	0.02° (0.00,0.03)
fast (exp-0.5)	0.00 (− 0.06,0.07)	0.01 (− 0.05,0.07)	−0.01 (− 0.05,0.03)	−0.01 (− 0.05,0.03)	−0.04° (− 0.08,0.01)	−0.04 (− 0.08,0.01)	0.01 (− 0.02,0.03)	0.01 (− 0.02,0.03)	0.02* (0.00,0.04)	0.02* (0.00,0.04)

^a^Model 1: Multiple linear mixed models adjusted for age, sex, BMI, current smoker and electrode location (random intercept model)

^b^Model 2: Multiple linear mixed models adjusted for age, sex, BMI, current smoker, electrode location, TST, N2(%), AHI and any medication (random intercept model). Statistically significant comparisons (*p* < 0.05) are in bold.: *p* < 0.1; *: *p* < 0.05; **: *p* < 0.01; ***: *p* < 0.001. The numbers in brackets correspond to the confidence interval

Lifetime diagnosis of SAD induced only a small decrease in the duration of fast spindles compared to the control group and no other spindle parameter was associated to this disorder. In contrast, people diagnosed with SAM showed differences in many parameters of the sleep spindles. These differences were generally in the opposite direction of those seen in the SZ group with an increase in slow spindle density, a decrease in fast spindle density, an increase in duration of all and slow spindles, a marked decrease in spindle frequency of all types of spindle as well as a tendency to decrease in the maximum spindle amplitude.

Compared to comparison probands, people with a lifetime diagnosis of BP-I and BP-II exhibited contrasting differences almost exclusively limited to spindle frequency (Table 2). The BP-I diagnosis is associated to a slight increase in all spindles and slow spindles frequencies, while BP-II diagnosis is associated with a weak decrease in all spindles and slow spindles frequencies. In addition, a marginal increase in the maximal amplitude of fast spindles was also associated to BP-II diagnosis compared to comparisons probands.

### Pairwise comparisons between schizophrenia group and people with schizoaffective and bipolar disorders

To determine whether differences in sleep spindle measures were specific for SZ, we performed pairwise comparisons of the different spindle parameters between the SZ group and each of the other psychiatric groups using a contrast testing framework from our multiple linear mixed model. Results were presented in Table 3. All and slow spindle densities were significantly lower in subjects with a diagnosis of SZ compared to those of people with a diagnosis of SAM, BP-I or BP-II. Following transformation of data, all and slow spindle durations in people with a lifetime diagnosis of SAM and BP-II were significantly lower to those observed in people with SZ. The decrease in duration of slow spindles in people with SZ compared to those with SAD or BP-I failed to reach statistical significance after adjustment for multiple testing. Duration of fast spindle was significantly higher only in people with a lifetime diagnosis of SAD. The frequency of all, slow and fast spindle exhibited significant differences only for the SAM diagnosis. No difference between SZ and the other psychiatric diagnosis was observed for the maximal amplitude of the spindles.

**Table 3:** Pairwise comparisons of sleep spindle measures between people with SZ and those with other diagnoses

	Comparisons											
	SZ vs SAD			SZ vs SAM			SZ vs BP-I			SZ vs BP-II		
	β^**a**^ (95CI)	***p-value***	***adj.***^***b***^ ***p-value***	β^**a**^ (95CI)	***p-value***	***adj.***^***b***^ ***p-value***	β^**a**^ (95CI)	***p-value***	***adj.***^***b***^ ***p-value***	β^**a**^ (95CI)	***p-value***	***adj.***^***b***^ ***p-value***
**Density**												
all	−0.46 (−1.33,0.41)	*0.300*	*0.300*	**−1.31** **(− 1.90,-0.72)**	***<.0001***	***< 0.001***	**− 1.25** **(− 2.15,-0.34)**	***0.007***	***0.014***	**− 1.29** **(− 2.08,-0.50)**	***0.001***	***0.004***
slow	−0.35 (− 1.06,0.37)	*0.341*	*0.341*	**− 1.39** **(− 1.89,-0.88)**	***<.0001***	***< 0.001***	**− 0.74** **(− 1.31,-0.17)**	***0.011***	***0.021***	**− 1.23** **(− 1.89,-0.58)**	***< 0.001***	***< 0.001***
fast (log)	− 0.44 (− 1.57,0.68)	*0.441*	*0.619*	−0.51 (− 1.42,0.41)	*0.279*	*0.619*	−1.02 (−2.03,-0.02)	*0.046*	*0.186*	−0.63 (− 1.60,0.34)	*0.206*	*0.619*
**Duration**												
all (log)	**−0.12** **(− 0.22,-0.02)**	***0.024***	***0.048***	**−0.23** **(− 0.32,-0.15)**	***<.0001***	***< 0.001***	**− 0.09** **(− 0.18,0.00)**	***0.044***	***0.048***	**−0.13** **(− 0.22,-0.04)**	***0.005***	***0.016***
slow (log)	−0.13 (− 0.24,-0.01)	*0.027*	*0.055*	**− 0.23** **(− 0.32,-0.14)**	***<.0001***	***< 0.001***	− 0.10 (− 0.20,-0.01)	*0.037*	*0.055*	**−0.14** **(− 0.23,-0.04)**	***0.006***	***0.019***
fast (exp-2.5)	0.56 (0.15,0.97)	*0.007*	***0.028***	0.37 (− 0.10,0.84)	*0.119*	*0.237*	0.44 (0.03,0.86)	*0.038*	*0.113*	0.26 (−0.16,0.68)	*0.232*	*0.237*
**Frequency**												
all	−0.13 (− 0.50,0.25)	*0.515*	*0.774*	**0.41** **(0.13,0.70)**	***0.004***	***0.017***	−0.13 (− 0.41,0.16)	*0.387*	*0.774*	0.28 (0.01,0.55)	*0.039*	*0.118*
slow	− 0.05 (− 0.23,0.13)	*0.614*	*0.614*	**0.18** **(0.05,0.31)**	***0.006***	***0.024***	−0.08 (− 0.20,0.04)	*0.179*	*0.357*	0.14 (0.02,0.26)	*0.024*	*0.073*
fast	−0.03 (− 0.17,0.12)	*0.708*	*1.000*	**0.17** **(0.05,0.30)**	***0.008***	***0.030***	0.03 (−0.10,0.16)	*0.641*	*1.000*	0.09 (−0.04,0.23)	*0.182*	*0.546*
**Max amplitude**												
all (exp-0.5)	0.02 (−0.05,0.10)	*0.571*	*1.000*	0.05 (−0.03,0.13)	*0.199*	*0.795*	0.00 (−0.07,0.07)	*0.923*	*1.000*	−0.01 (− 0.07,0.06)	*0.875*	*1.000*
slow (exp-0.5)	0.02 (−0.05,0.10)	*0.536*	*1.000*	0.05 (−0.03,0.13)	*0.195*	*0.780*	0.00 (−0.07,0.07)	*0.909*	*1.000*	−0.01 (− 0.07,0.06)	*0.882*	*1.000*
fast (exp-0.5)	0.02 (−0.05,0.10)	*0.548*	*1.000*	0.05 (−0.03,0.13)	*0.258*	*1.000*	0.00 (−0.07,0.07)	*0.972*	*1.000*	−0.01 (− 0.08,0.06)	*0.786*	*1.000*

^a^Contrast testing framework from multiple linear mixed models adjusted for age, sex, BMI, current smoker, electrodes location, TST, N2 (%), AHI and any medication (random intercept model)

^b^Adjusted *p*-values for multiple testing using stepwise Bonferroni-Holm

## Discussion

The pilot study presented herein provides novel information on the association of various psychotic disorders with sleep spindle parameters in a general population-based cohort. Our results highlight the decrease in all spindle density and duration parameters as biomarkers of schizophrenia and indicate different sleep spindle phenotype in participants with schizoaffective disorders. Furthermore, our results suggest that the spindle (slow and fast) densities were more elevated in people with a history of maniac symptoms (SAM, BP-I and BP-II) than in those without (SZ, SAD).

In an original way, this study presents some specificities in terms of target population and method of sleep recording. Indeed, while most studies on the effects of psychiatric disorders on sleep spindles have been carried out on clinical cohorts of in- and out-patients selected by diagnosis, our study was performed in people from the community. Participants were thus older (about 60 years) than in most other studies investigating sleep spindles, which are known to have lower density and duration when age increases [41, 42]. Our linear regression models used to compare diagnosis effects on spindle parameters include this parameter even if age distribution was not significantly different between diagnoses (Table 1).

Another interesting specificity of our study is that sleep EEG recordings have been performed “at home” with an ambulatory system while most of other studies were realized in hospital or in sleep laboratory. Such more “naturalistic” recording conditions reduced various stress factors such as a new environment as well as the psychological impact of being observed during sleep. Although sleep with an ambulatory system does not exactly reflect usual nighttime sleep, this technique has been described as reducing the “first-night-effect” usually observed following the first night of recordings at hospital and characterized by a reduction in sleep quality [43–45]. This constitutes a relevant issue regarding the expected sensitivity of people with psychiatric disorders to the various stressors involved in “first-night-effect”.

These specificities of our study may explain why no significant difference in sleep variables between participants with and without psychiatric diagnosis was observed (Supplemental Table 4). Indeed, this is unexpected since sleep disturbances are usually associated to schizophrenia spectrum disorders [46–48] as well as to bipolar disorders [49, 50] in clinical cohorts. However, as suggested by previous study with HypnoLaus cohort [51], 30–45% of subjects without psychiatric diagnosis may have some sleep disturbances, regardless of the severity of the disturbances. Hence, comparisons of sleep parameters between SZ, SAD, SAM, BP-I, BP-II groups and the control group likely cannot give significant difference.

The analysis of sleep spindle parameters provides new information on their phenotypes in different psychotic disorders. First of all, in agreement with other studies in schizophrenia (see review [52]), we showed that people with a lifetime diagnosis of schizophrenia displayed a significant decrease in spindle density and duration when compared to other groups (Fig. 1; Table 2). Curiously, this reduction in spindle density observed was significant for all (slow and fast) and for slow spindles but not for fast spindles for which, only a tendency was present (Table 2). This may suggest a differential effect of lifetime diagnosis of SZ on the different thalamo-cortical circuits at the origin of the fast and slow spindles. Previous studies reported a decrease in sleep spindles at early stage of psychosis in medicated as well as naïve patients [26, 53–55]. Interestingly, since people with schizophrenia were in an older age-range (63.2 ± 12.6y) in our study, our data suggest a persistence of spindle deficit throughout life, even in long term stabilized patients. Thus, our results strengthen the idea that sleep spindles deficit could constitute an early and persistent biomarker of schizophrenia (i.e. an endophenotype). Spindle deficits in healthy first-degree relatives [56] and genetic studies showing an association of the gene CACNA1I (that encode for the subunit Ca_V_3.3 channel) with schizophrenia [57, 58], also support the hypothesis of a genetic risk factor involved in the thalamo-cortical system deficiency at the basis of spindle alteration.

Compared to the other groups, spindle frequency was the main parameter affected in bipolar I and -II disorders (Table 2). Only a small but significant increase in fast spindle amplitude is the other change observed in the BP-II group. Contrary to Ritter et al. who reported a decrease in density and frequency of fast spindles in frontal and central derivations [33], our results indicated that only frequency of all spindles and slow spindles were changed in BP-I or BP-II groups. However, these discrepancies should be treated with caution since Ritter and collaborators considered fast spindle when intra-spindle frequency was > 13 Hz while we applied a cutoff at ≥14 Hz. Thus, we reported a mean value of 13.09 Hz for slow-spindles in BP-I group and of 13.26 Hz in BP-II group, both considered as fast-spindles in Ritter’s study. Noteworthy, in BP-I and BP-II groups, spindle frequency changed in opposite directions (Table 2). These opposite variations likely participate also to the difference between our results and those in which participants with BP-I and BP-II were not differentiated [32, 33]. This heterogeneity in sleep spindle parameters is consistent with that reported between bipolar disorders sub-types in term of clinical features, cognition and brain structure [59–61] as well as susceptibility to sleep disturbances [62].

To the best of our knowledge, our study is the first to investigate the sleep spindles in people with a diagnosis of sub-types of schizoaffective disorders. Compared to other participants, densities of the slow and fast spindles are respectively increased and decreased in SAM group. Duration of all and slow spindles were higher and frequency of all types of spindles was decreased. Although these effects are statistically significant, the small number of participants in this group should prompt us to be cautious about the interpretation of these results. In SAD group, only duration of fast spindles were slightly decreased (Table 2). Consequently, with regard to the sleep spindle phenotype, schizoaffective disorders seem to be more heterogeneous than BP-I and BP-II disorders. In view of the specific alterations in sleep spindles across diagnostic groups, our results may suggest that a history of manifestations of mania or hypomania leaves a “footprint” on the sleep spindles characterized by an alteration in their frequency rather than in their density. However, since direction of frequency changes in these three groups was different (i.e. increase for BP-I and decrease for SAM and BP-II), the impact of mania on sleep spindle parameter remains difficult to explain.

Comparisons of participants with schizophrenia with those having other diagnoses showed divergent results for spindle duration, frequency and amplitude (Table 3). However, considering the density of sleep spindles (all- and slow-spindles), people with SAD cannot be considered as different from those with a schizophrenia diagnosis (Table 3). Given the role of the thalamo-cortical circuits in the genesis and modulation of sleep spindles, our results might suggest the existence of different disturbances of these circuits in schizophrenia and SAD on one hand and in SAM and bipolar disorders on the other. Some differences between SAD and SAM sub-types have been already reported in a longitudinal study in which neuropsychological evaluation and functional magnetic resonance imaging (fMRI) were performed during episodes or following remission [63]. Results showed that remitted people with SAD exhibit persistent impairments of memory and have dysfunction of the default mode network similar to people with schizophrenia, whereas remitted people with SAM improve their memory and normalize fMRI signal in medial frontal gyrus suggesting a deeper and more lasting impairment of brain networks in people with SAD as compared to those with SAM.

Our results could also have some implications in term of cognitive differences between diagnoses. Indeed, sleep spindles are thought to play a key role in sleep-related memory mechanisms [17] and their number are positively correlated to cognitive abilities in healthy people as well as in schizophrenia patients [11, 27]. Therefore, we could expect that memory was less impaired in participants with diagnosis of SAM, BP-I, and BP-II than in participants with schizophrenia and SAD. Although a recent meta-analysis indicates that schizophrenia patients have more severe cognitive impairments than bipolar patients [64], no evidence of difference in sleep-related memory consolidation between these diagnosis groups were available.

Our study has several limitations. First, due to the low prevalence of these disorders in general population, the number of participants with a schizophrenia spectrum and bipolar disorders was small. Accordingly, statistical power was low and associations that did not have a large effect size could not be identified. In addition, although we still found significant differences after correcting for multiple testing, the risk of false positive cannot be ruled out, particularly in the SAM group. Second, the cross-sectional nature of the data impeded us to draw conclusions regarding the directions of the observed associations. Third, the measurement of sleep spindles relied on only four electrodes. This was likely to lead to an underestimation of fast-spindles and impeded us from precisely localizing effects related to diagnoses. Such a technical limitation is inherent to ambulatory PSG recordings that enabled sleep parameters determination in a population-based sample.

Taking into account all these limitations, our pilot study, using with an original way a general population-based cohort, brought new elements that argued in favor of a deficit of sleep spindles density and duration in people with schizophrenia. In addition, while we could expect a gradual change in intensity of the same sleep spindle parameters through psychotic diagnoses, our results seem to indicate a more complex situation in which the frequency of sleep spindles might be more impacted by diagnoses including a history of mania or hypomania manifestations. Since our study included a small number of participants with such a psychiatric diagnosis, further studies are required to confirm these effects and to determine underpinning mechanisms.

## Supplementary Information

**Additional file 1: Supplement 1.** Polysomnographic recordings and determination of the sleep variables. **Supplement 2.** Definition and analyze of sleep parameters measured by PSG. **Supplement 3.** Statistical methods. **Supplementary Table 1.** Sleep variables assessed by polysomnography in each group. **Supplementary Table 2.** Sleep spindle parameters at F3 and F4 electrodes by lifetime diagnosis. **Supplementary Table 3.** Sleep spindle parameters at C3 and C4 by lifetime diagnosis. **Supplementary Table 4.** Spearman’s rho correlation coefficients of spindle parameters between electrodes.

## Data Availability

Non-identifiable individual-level data are available for researchers who seek to answer questions related to health and disease in the context of research projects who meet the criteria for data sharing by research committees. Please follow the instructions at https://www.colaus-psycolaus.ch for information on how to submit an application for gaining access to CoLaus|PsyCoLaus data.

## Abbreviations

AHI: Apnea/hypopnea index
BMI: Body mass index
BP-I: Bipolar disorder type I
BP-II: Bipolar type-II
CA1: Region 1 of hippocampal Ammon’s horn
CACNA1I: Calcium Voltage-Gated Channel Subunit Alpha1 I
DIGS: Diagnostic interview for genetic studies
DSM-5: Diagnostic and statistical manual of mental disorders, Fifth edition
EEG: Electro-encephalogram
FU: Follow-up
N2: Stage 2 of NREM sleep
NREM: Non-REM sleep
PSG: Polysomnography
REM: Rapid eye movements
SAD: Schizoaffective depressive subtype
SAM: Schizoaffective manic subtype
SZ: Schizophrenia
TC: Thalamo-cortical neurons
TRN: Thalamic reticular nucleus
TST: Total sleep time
