# Supplementary material for: Sleep spindles in people with schizophrenia, schizoaffective disorders or bipolar disorders: a pilot study in a general population-based cohort
Source: BMC Psychiatry. 2022 Dec 3;22:758. doi: 10.1186/s12888-022-04423-y (PMC9719140; doi:10.1186/s12888-022-04423-y)
Supplement: Supplementary file 1 — Additional file 1: Supplement 1. Polysomnographic recordings and determination of the sleep variables. Supplement 2. Definition and analyze of sleep parameters measured by PSG. Supplement 3. Statistical methods. Supplementary Table 1. Sleep variables assessed by polysomnography in each group. Supplementary Table 2. Sleep spindle parameters at F3 and F4 electrodes by lifetime diagnosis. Supplementary Table 3. Sleep spindle parameters at C3 and C4 by lifetime diagnosis. Supplementary Table 4. Spearman’s rho correlation coefficients of spindle parameters between electrodes. [file 12888_2022_4423_MOESM1_ESM.docx]

**Additional file 1**

**Sleep spindle alterations in people with schizophrenia, schizoaffective disorders or bipolar disorders in a general population-based cohort**

**Jean-Marie Petit PhD.^1^, Marie-Pierre F. Strippoli Msc.^2^, Serateh Ranjbar PhD.^2^, Aurélie Stephan PhD.^3^, José Haba-Rubio MD.^3^, Geoffroy Solelhac MD.^3^, Raphaël Heinzer MD, MPH.^3^, Martin Preisig MD., MPH. ^2^ , Francesca Siclari MD^3^ and Kim Q. Do PhD.^1^**

1-Center for Psychiatric Neuroscience (CNP), Department of Psychiatry, Lausanne University Hospital and University of Lausanne, CH-1008 Prilly, Switzerland.

2- Center for Psychiatric Epidemiology and Psychopathology (CEPP), Department of Psychiatry, Lausanne University Hospital and University of Lausanne, CH-1008 Prilly, Switzerland

3-Center for Sleep Research and Investigation (CIRS), Lausanne University Hospital and University of Lausanne, Lausanne, CH-1009 Lausanne, Switzerland.

**Supplementary information**

- Supplement 1: Polysomnographic recordings and determination of the sleep variables.
- Supplement 2: Definition and analyze of sleep parameters measured by PSG.
- Supplement 3: Statistical methods.
- Legend Supplementary Table 1
- Legend Supplementary Table 2
- Legend Supplementary Table 3
- Legend Supplementary Table 4
- Supplementary Table 1: Sleep variables assessed by polysomnography in each group
- Supplementary Table 2: Sleep spindle parameters at F3 and F4 electrodes by lifetime diagnosis.
- Supplementary Table 3: Sleep spindle parameters at C3 and C4 by lifetime diagnosis.
- Supplementary Table 4: Spearman's rho correlation coefficients of spindle parameters between electrodes.

**Supplement 1: Polysomnographic recordings and determination of the sleep variables.**

A full night ambulatory PSG was performed using a portable sleep/wake recording system (EMBLA Titanium®, Embla systems, Inc., Broomfield, CO, USA). Electrodes were placed at CIRS (Center for Investigation and Research in Sleep, Lausanne University Hospital, Lausanne, Switzerland) by a trained technician between 17h and 20h. Electroencephalogram (EEG), was recorded from frontal, central, and occipital areas (F3-M2, F4-M1, C3-M2, C4-M1, O1-M2, O2-M1) according to the international 10/20 electrodes configuration system. Right and left electro-oculogram (EOG), submental electromyogram (EMG), right and left leg EMG, thoracic and abdominal breathing movements (inductance plethysmography), electrocardiography, pulse oximetry, respiratory airflow via nasal cannula connected to a pressure transducer, and body position were also recorded. Participants were instructed to follow their habitual sleep habits.

All PSG recordings were visually scored by two trained sleep technicians using Somnologica software (Version 5.1.1, by Embla Flaga, Reykjavik, Iceland) and reviewed by a trained sleep physician (JHR). Random quality checks were performed by a second physician (RH). Quality control for concordance rate between the two PSG scorers was implemented periodically to ensure that both scorers achieved at least a 90% level of agreement for sleep stages and respiratory events and an 85% level of agreement for arousals. Sleep stages, leg movements, and arousals were scored according to the 2007 American Academy of Sleep Medicine (AASM) criteria. Apneas/hypopneas were scored according to the AASM 2013 rules.

**Supplement 2: Definition of sleep parameters measured by polysomnography.**

Following scoring of polysomnographic recordings, the classical sleep parameters have been calculated according to the following definitions:

-Total sleep time (TST) = Time spent in any stage of sleep from sleep onset to morning awakening (in minutes).

-Total Rest Time (TRT) = Time spent in bed between going to bed and getting up (in minutes).

-Sleep Efficiency (SE) = ratio between the total sleep time and time spent in bed (in percent).

-Sleep Onset Latency (SOL) (also referred as N1 latency) = time between going to bed and the first stage of sleep.

REM Onset latency (REMOL) = Latency to first REM stage after sleep onset.

-Wake after sleep onset (WASO) = total time awake following the first stage of sleep (in minutes).

-Stage 1 (N1) = total time spent in stage 1 of Non-REM sleep (in minutes).

-Stage N1% = percentage of TST spent in N1 sleep stage (in percent).

-Stage 2 (N2) = total time spent in stage 2 of Non-REM sleep (in minutes).

-Stage N2% = percentage of TST spent in N2 sleep stage (in percent).

-Slow Wave Sleep or Stage 3 (N3) = total time spent in stage 3 of Non-REM sleep (in minutes).

-Stage N3% = percentage of TST spent in N3 sleep stage (in percent).

-Rapid Eye Movement Sleep (REM) = total time spent in REM sleep stage (in minutes).

-Stage REM% = percentage of TST spent in REM sleep stage (in percent).

-Periodic Leg Mouvement Index (PLMI) = number of events / hour of sleep.

-Respiratory Events Related Arousal Index (RERAI) = number of all respiratory events / hour of sleep.

-Total Arousal Index (TotArousI) = Total number of arousals (scored according to the 2007 American Academy of Sleep Medicine (AASM) criteria) / hour of sleep.

-Apnea/Hypopnea Index (AHI) = Total number of apnea and hypopnea (scored according to the AASM 2013 rules) / hour of sleep.

-Mean pulse Oxygen Saturation (SpO2 mean) = Mean pulse oxygen saturation calculated during TST at the tip of index (in percent).

-4% oxygen desaturation index (odi4) = number of decreases in SpO2 ≥4%from baseline / hour of sleep.

**Supplement 3: Statistical methods.**

-Contrast testing framework

Contrast testing framework on the fitted models was used to test whether participants with SAD, SAM, BP-I or BP-II differed from the participants with SZ. Contrast testing framework is a common practice in the multiple hypothesis testing, where each hypothesis is formulated as a contrast (linear combination) of different coefficients in the model. Hence, contrast is a encoding of the research question. Here in this all the pairwise comparisons that were of interest are encoded as a contrast.

-Bonferroni-Holm adjustment

Since we performed several tests, we expect to find significant results even if all the null hypotheses are true. Control of this can be achieved with family error rate (FWER) or false discovery rate (FDR) control procedures. Here we performed a Bonferroni-Holm adjustment that belongs to the FWER class of procedure. This correction is a less conservative and uniformly more powerful correction than the Bonferroni correction. In addition, by controlling the FWER, this setting automatically controls the FDR. The Bonferroni-Holm test does this by correcting p-values sorted from smallest to largest, and the algorithm used here stopped when it reached the first non-rejected hypothesis.

**Legend of Supplementary Table 1:** **Sleep variables assessed by polysomnography in each group**

SZ: Schizophrenia; SAD; Schizoaffective disorder - depressive type; SAM; Schizoaffective disorder - bipolar type; BP-I: Bipolar type I disorder; BP-II: Bipolar type II disorder; CTL: comparison participants. TST: Total sleep time; TRT: Total recording time; SE: Sleep efficiency; SOL: Sleep onset latency; REMOL: REM onset latency; N1: NREM stage 1; N2: NREM stage 2; N3: NREM stage 3; REM: Rapid eye movement sleep; WASO: Wake after sleep onset; PLMI: Periodic limb movement index; RERAI: Respiratory events related arousal index; TotArousI: Total arousal index; SpO2: Mean pulse oxygen saturation; odi4: 4% oxygen desaturation index; min: minute; hr: hour. For each parameter mean is given and (lower-upper) 95% confidence interval is indicated under the mean in italic. Kruskall-Wallis test (non-parametric ANOVA), *: p<0.05.

**Legend of Supplementary Table 2:** **Sleep spindle parameters at F3 and F4 electrodes by lifetime diagnosis.**

SZ: schizophrenia; SAD; schizoaffective disorder - depressive type; SAM; schizoaffective disorder - bipolar type; BP-I: bipolar type I disorder; BP-II: bipolar type II disorder; CTL: comparison participants; sd: standard deviation; IQR: interquartile range.

**Legend of Supplementary Table 3:** **Sleep spindle parameters at C3 and C4 by lifetime diagnosis.**

SZ: schizophrenia; SAD; schizoaffective disorder - depressive type; SAM; schizoaffective disorder - bipolar type; BP-I: bipolar type I disorder; BP-II: bipolar type II disorder; CTL: comparison participants; sd: standard deviation; IQR: interquartile range.

**Legend of Supplementary Table 4: Spearman's rho correlation coefficients of spindle parameters between electrodes.**

rs: spearman's rho correlation coefficient. >0.80 very strong; 0.60-0.80: moderate; 0.30-0.60: fair.

**Supplementary Table 1: Sleep variables assessed by polysomnography in each group**

| **Groups** | **SZ** | **SAD** | **SAM** | **BP-I** | **BP-II** | **CTL** |
| --- | --- | --- | --- | --- | --- | --- |
| Number | 7 | 12 | 4 | 17 | 9 | 988 |
| **Sleep Variables** |  |  |  |  |  |  |
| TST (min) | 373 | 386 | 328 | 398 | 404 | 395 |
|  | *(255-491)* | *(336-436)* | *(254-403)* | *(348-448)* | *(349-460)* | *(390-399)* |
| **TRT (min)*** | **529** | **524** | **411** | **483** | **508** | **489** |
|  | ***(472-586)*** | ***(485-563)*** | ***(353-469)*** | ***(445-521)*** | ***(449-567)*** | ***(485-493)*** |
| SE (%) | 73.0 | 78.8 | 83.0 | 84.5 | 83.2 | 83.9 |
|  | *(57.4-88.7)* | *(69.7-87.8)* | *(61.3-105.0)* | *(78.6-90.5)* | *(74.0-92.3)* | *(83.2-84.6)* |
| SOL (min) | 29 | 31 | 13 | 17 | 19 | 16 |
|  | *(0-66)* | *(1-61)* | *(1-27)* | *(4-30)* | *(10-28)* | *(15-17)* |
| REMOL (min) | 107 | 114 | 92 | 97 | 106 | 87 |
|  | *(26-189)* | *(56-173)* | *(9-176)* | *(66-129)* | *(50-162)* | *(87-94)* |
| N1 (min) | 75 | 47 | 32 | 46 | 54 | 47 |
|  | *(30-119)* | *(36-59)* | *(19-45)* | *(37-54)* | *(31-76)* | *(46-49)* |
| N1 (% of TST) | 21.3 | 12.4 | 10.1 | 11.8 | 13.8 | 12.2 |
|  | *(10.8-31.9)* | *(9.1-15.8)* | *(3.5-16.8)* | *(9.7-14)* | *(8-19.7)* | *(11.8-12.7)* |
| N2 (min) | 179 | 160 | 141 | 183 | 183 | 185 |
|  | *(74-284)* | *(130-191)* | *(74-209)* | *(141-226)* | *(151-214)* | *(182-189)* |
| N2 (% of TST) | 45.4 | 41.6 | 43.1 | 44.6 | 45.5 | 46.9 |
|  | *(33.5-57.3)* | *(35.6-47.6)* | *(23.9-62.3)* | *(38.4-50.9)* | *(38.4-52.7)* | *(46.2-47.5)* |
| N3 (min) | 54 | 94 | 86 | 77 | 79 | 75 |
|  | *(38-71)* | *(68-120)* | *(50-122)* | *(62-92)* | *(49-109)* | *(73-77)* |
| N3 (% of TST) | 15.6 | 24.2 | 25.9 | 20.1 | 19 | 19.1 |
|  | *(9.3-22.6)* | *(18.6-29.8)* | *(19.2-32.6)* | *(16-24.2)* | *(13.4-24.6)* | *(18.6-19.6)* |
| REM (min) | 65 | 85 | 69 | 92 | 89 | 87 |
|  | *(37-92)* | *(69-100)* | *(17-121)* | *(78-106)* | *(66-113)* | *(85-89)* |
| REM (%) | 17.4 | 21.7 | 20.8 | 23.4 | 21.7 | 21.8 |
|  | *(11.7-23)* | *(19.4-24.1)* | *(7.5-34.2)* | *(20.6-26.3)* | *(17.5-25.8)* | *(21.4-22.1)* |
| WASO (min) | 127 | 107 | 69 | 68 | 85 | 78 |
|  | *(66-187)* | *(58-156)* | *(0-160)* | *(46-91)* | *(34-136)* | *(74-82)* |
| PLMI (#event/hr) | 42.6 | 25 | 11.9 | 18.2 | 11.3 | 14.8 |
|  | *(10.4-74.8)* | *(8.0-42.0)* | *(0-47.1)* | *(5.78-30.6)* | *(0-28)* | *(13.2-16.4)* |
| RERAI (#event/hr) | 0.04 | 0.4 | 1.13 | 1.61 | 1.7 | 1.06 |
|  | *(0-0.09)* | *(0.08-0.72)* | *(0-3.30)* | *(0.45-2.77)* | *(0.42-2.98)* | *(0.95-1.16)* |
| TotArousI (#event/hr) | 29.9 | 17 | 15.8 | 18.8 | 21.7 | 22.4 |
|  | *(10.6-49.3)* | *(13.4-20.6)* | *(0.4-31.2)* | *(15.8-21.8)* | *(12.8-30.6)* | *(21.7-23.1)* |
| SpO2 (mean) | 91.5 | 94.1 | 93.3 | 93.6 | 94.2 | 93.9 |
|  | *(87.2-95.8)* | *(93.5-94.6)* | *(90.7-95.9)* | *(92.7-94.5)* | *(93.4-94.9)* | *(93.7-94.1)* |
| odi4 (/hr) | 21.8 | 4.58 | 5.55 | 8.52 | 7.41 | 9.21 |
|  | *(0-47.60)* | *(1.84-7.33)* | *(0-13.20)* | *(4.47-12.60)* | *(1.62-13.20)* | *(8.41-10.00)* |

**Supplementary Table 2: Sleep spindle parameters at F3 and F4 electrodes by lifetime diagnosis.**

|  |  |  |  | **Lifetime diagnosis** | | | | | | | | | | | |
| --- | --- | --- | --- | --- | --- | --- | --- | --- | --- | --- | --- | --- | --- | --- | --- |
|  |  | **ALL** | | **SZ** | | **SAD** | | **SAM** | | **BP-I** | | **BP-II** | | **CTL** | |
|  | **N** | **mean (sd)** | **median (IQR)** | **mean (sd)** | **median (IQR)** | **mean (sd)** | **median (IQR)** | **mean (sd)** | **median (IQR)** | **mean (sd)** | **median (IQR)** | **mean (sd)** | **median (IQR)** | **mean (sd)** | **median (IQR)** |
| **F3** |  |  |  |  |  |  |  |  |  |  |  |  |  |  |  |
| Density |  |  |  |  |  |  |  |  |  |  |  |  |  |  |  |
| all | 1032 | 2.45 (1.39) | 2.24 (1.96) | 1.14 (0.66) | 1.33 (1.24) | 1.83 (1.06) | 1.88 (1.61) | 3.02 (0.74) | 2.91 (1.07) | 2.45 (1.75) | 2.30 (1.99) | 2.99 (1.50) | 3.21 (1.60) | 2.45 (1.38) | 2.24 (1.96) |
| slow | 1031 | 0.19 (0.24) | 0.11 (0.17) | 0.10 (0.11) | 0.07 (0.13) | 0.36 (0.51) | 0.14 (0.46) | 0.53 (0.25) | 0.56 (0.39) | 0.14 (0.16) | 0.07 (0.20) | 0.29 (0.34) | 0.14 (0.36) | 0.19 (0.24) | 0.11 (0.17) |
| fast | 1021 | 2.26 (1.31) | 2.05 (1.80) | 1.04 (0.62) | 1.25 (1.11) | 1.53 (0.71) | 1.68 (1.21) | 2.48 (0.71) | 2.19 (0.87) | 2.33 (1.72) | 2.25 (1.84) | 2.70 (1.38) | 2.69 (1.28) | 2.27 (1.30) | 2.05 (1.80) |
| Duration |  |  |  |  |  |  |  |  |  |  |  |  |  |  |  |
| all | 1032 | 0.90 (0.16) | 0.87 (0.19) | 0.84 (0.17) | 0.82 (0.23) | 1.01 (0.31) | 0.92 (0.43) | 1.05 (0.14) | 1.00 (0.18) | 0.88 (0.13) | 0.88 (0.16) | 0.93 (0.14) | 0.92 (0.12) | 0.90 (0.16) | 0.87 (0.19) |
| slow | 1031 | 1.08 (0.23) | 1.05 (0.26) | 1.02 (0.16) | 1.04 (0.22) | 1.07 (0.24) | 1.06 (0.20) | 1.31 (0.17) | 1.25 (0.20) | 1.07 (0.19) | 1.11 (0.34) | 1.10 (0.21) | 1.13 (0.30) | 1.07 (0.23) | 1.05 (0.26) |
| fast | 1021 | 0.89 (0.16) | 0.85 (0.18) | 0.82 (0.17) | 0.74 (0.22) | 0.98 (0.30) | 0.91 (0.33) | 0.99 (0.14) | 0.93 (0.18) | 0.87 (0.13) | 0.88 (0.21) | 0.91 (0.13) | 0.89 (0.15) | 0.88 (0.16) | 0.85 (0.18) |
| Frequency |  |  |  |  |  |  |  |  |  |  |  |  |  |  |  |
| all | 1032 | 13.10 (0.34) | 13.11 (0.45) | 13.15 (0.44) | 12.94 (0.60) | 13.16 (0.54) | 13.26 (0.67) | 12.64 (0.13) | 12.67 (0.20) | 13.22 (0.40) | 13.20 (0.43) | 12.92 (0.23) | 12.94 (0.32) | 13.10 (0.33) | 13.11 (0.45) |
| slow | 1031 | 11.78 (0.06) | 11.78 (0.07) | 11.79 (0.10) | 11.82 (0.20) | 11.76 (0.07) | 11.78 (0.07) | 11.77 (0.07) | 11.77 (0.13) | 11.79 (0.07) | 11.80 (0.05) | 11.77 (0.07) | 11.75 (0.11) | 11.78 (0.06) | 11.78 (0.07) |
| fast | 1021 | 13.20 (0.29) | 13.18 (0.38) | 13.26 (0.41) | 13.25 (0.56) | 13.28 (0.44) | 13.37 (0.55) | 12.83 (0.08) | 12.85 (0.10) | 13.29 (0.38) | 13.20 (0.40) | 13.04 (0.16) | 13.04 (0.22) | 13.20 (0.29) | 13.18 (0.38) |
| Amplitude |  |  |  |  |  |  |  |  |  |  |  |  |  |  |  |
| all | 1032 | 14.99 (23.3) | 10.71 (4.5) | 17.19 (19.4 | 10.33 (8.7) | 20.87 (22.4) | 10.32 (9.6) | 16.65 (7.9) | 14.67 (10.5) | 13.26 (10.9) | 9.67 (6.7) | 10.29 (1.7) | 10.29 (2.3) | 14.97 (23.6) | 10.75 (4.5) |
| slow | 1031 | 15.71 (25.9) | 10.96 (4.7) | 15.86 (16.1) | 11.43 (4.1) | 16.62 (18.3) | 9.95 (6.0) | 15.91 (6.2) | 14.30 (8.0) | 15.25 (13.0) | 11.16 (9.3) | 10.96 (2.1) | 11.25 (2.5) | 15.75 (26.4) | 10.96 (4.6) |
| fast | 1021 | 14.94 (23.4) | 10.70 (4.4) | 17.24 (19.6) | 10.29 (8.9) | 20.97 (22.4) | 10.23 (10.6) | 16.69 (8.12) | 14.69 (10.9) | 13.16 (10.6) | 9.54 (6.7) | 10.24 (1.6) | 10.25 (2.3) | 14.92 (23.7) | 10.72 (4.4) |
| **F4** |  |  |  |  |  |  |  |  |  |  |  |  |  |  |  |
| Density |  |  |  |  |  |  |  |  |  |  |  |  |  |  |  |
| all | 1030 | 2.44 (1.41) | 2.25 (1.93) | 1.10 (0.74) | 1.04 (1.60) | 2.00 (1.18) | 1.85 (1.82) | 2.91 (0.75) | 2.61 (0.98) | 2.33 (1.81) | 2.25 (1.90) | 3.03 (1.38) | 3.10 (0.79) | 2.45 (1.41) | 2.25 (1.94) |
| slow | 1029 | 0.19 (0.24) | 0.11 (0.18) | 0.14 (0.20) | 0.05 (0.02) | 0.30 (0.39) | 0.14 (0.46) | 0.50 (0.26) | 0.41 (0.37) | 0.10 (0.12) | 0.07 (0.11) | 0.35 (0.40) | 0.16 (0.46) | 0.19 (0.24) | 0.11 (0.17) |
| fast | 1023 | 2.26 (1.32) | 2.05 (1.78) | 1.00 (0.65) | 1.02 (1.24) | 1.75 (0.90) | 1.70 (1.30) | 2.41 (0.54) | 2.30 (0.80) | 2.23 (1.78) | 2.19 (1.95) | 2.68 (1.25) | 2.83 (0.88) | 2.27 (1.32) | 2.06 (1.79) |
| Duration |  |  |  |  |  |  |  |  |  |  |  |  |  |  |  |
| all | 1030 | 0.90 (0.17) | 0.87 (0.19) | 0.82 (0.12) | 0.80 (0.25) | 0.99 (0.26) | 0.91 (0.40) | 1.05 (0.11) | 1.02 (0.14) | 0.94 (0.16) | 0.93 (0.22) | 0.95 (0.14) | 0.90 (0.19) | 0.90 (0.16) | 0.87 (0.19) |
| slow | 1029 | 1.06 (0.22) | 1.04 (0.26) | 0.98 (0.20) | 0.91 (0.34) | 1.08 (0.32) | 1.02 (0.47) | 1.34 (0.19) | 1.25 (0.20) | 1.05 (0.34) | 1.01 (0.28) | 1.12 (0.18) | 1.12 (0.21) | 1.06 (0.22) | 1.04 (0.26) |
| fast | 1023 | 0.88 (0.16) | 0.85 (0.18) | 0.81 (0.11) | 0.79 (0.17) | 0.97 (0.26) | 0.89 (0.34) | 0.99 (0.12) | 0.97 (0.16) | 0.93 (0.15) | 0.93 (0.22) | 0.92 (0.15) | 0.86 (0.22) | 0.88 (0.16) | 0.85 (0.17) |
| Frequency |  |  |  |  |  |  |  |  |  |  |  |  |  |  |  |
| all | 1030 | 13.12 (0.34) | 13.12 (0.45) | 13.15 (0.43) | 12.96 (0.79) | 13.22 (0.59) | 13.16 (0.80) | 12.70 (0.06) | 12.72 (0.07) | 13.23 (0.38) | 13.19 (0.48) | 12.86 (0.19) | 12.87 (0.24) | 13.12 (0.33) | 13.12 (0.44) |
| slow | 1029 | 11.78 (0.06) | 11.78 (0.06) | 11.75 (0.07) | 11.79 (0.10) | 11.74 (0.15) | 11.76 (0.06) | 11.75 (0.04) | 11.75 (0.06) | 11.79 (0.05) | 11.79 (0.05) | 11.79 (0.06) | 11.78 (0.08) | 11.78 (0.05) | 11.78 (0.06) |
| fast | 1023 | 13.21 (0.30) | 13.19 (0.40) | 13.24 (0.35) | 13.07 (0.54) | 13.31 (0.52) | 13.24 (0.71) | 12.89 (0.08) | 12.87 (0.11) | 13.29 (0.37) | 13.22 (0.54) | 12.99 (0.12) | 12.97 (0.20) | 13.21 (0.29) | 13.20 (0.39) |
| Amplitude |  |  |  |  |  |  |  |  |  |  |  |  |  |  |  |
| all | 1030 | 15.71 (24.8) | 10.81 (4.3) | 17.75 (19.5) | 10.77 (11.1) | 20.37 (21.5) | 9.86 (9.3) | 16.54 (7.8) | 14.54 (9.9) | 30.55 (79.3) | 9.83 (5.0) | 10.55 (1.8) | 10.90 (2.4) | 15.42 (23.1) | 10.86 (4.3) |
| slow | 1029 | 16.02 (26.5) | 11.12 (4.5) | 18.53 (15.2) | 13.07 (3.9) | 17.58 (13.6) | 10.27 (9.1) | 16.64 (6.67) | 15.45 (9.6) | 39.77 (111) | 10.86 (7.4) | 11.01 (1.7) | 11.32 (2.6) | 15.61 (22.8) | 11.12 (4.5) |
| fast | 1023 | 15.63 (24.5) | 10.76 (4.3) | 17.91 (19.7) | 10.81 (11.8) | 20.32 (21.6) | 9.80 (9.5) | 16.52 (8.0) | 14.42 (10.0) | 29.38 (74.6) | 9.84 (5.2) | 10.45 (1.8) | 10.67 (2.3) | 15.37 (23.1) | 10.79 (4.3) |

**Supplementary Table 3: Sleep spindle parameters at C3 and C4 electrodes by lifetime diagnosis.**

|  |  |  |  | **Lifetime diagnosis** | | | | | | | | | | | |
| --- | --- | --- | --- | --- | --- | --- | --- | --- | --- | --- | --- | --- | --- | --- | --- |
|  |  | **ALL** | | **SZ** | | **SAD** | | **SAM** | | **BP-I** | | **BP-II** | | **CTL** | |
|  | **N** | **mean (sd)** | **median (IQR)** | **mean (sd)** | **median (IQR)** | **mean (sd)** | **median (IQR)** | **mean (sd)** | **median (IQR)** | **mean (sd)** | **median (IQR)** | **mean (sd)** | **median (IQR)** | **mean (sd)** | **median (IQR)** |
| **C3** |  |  |  |  |  |  |  |  |  |  |  |  |  |  |  |
| Density |  |  |  |  |  |  |  |  |  |  |  |  |  |  |  |
| all | 1025 | 2.56 (1.54) | 2.39 (2.25) | 1.43 (1.28) | 1.46 (1.58) | 2.22 (1.27) | 2.50 (1.75) | 2.45 (1.19) | 2.48 (1.55) | 2.45 (1.79) | 2.15 (2.39) | 2.49 (1.15) | 2.79 (0.76) | 2.58 (1.55) | 2.39 (2.26) |
| slow | 1024 | 0.06 (0.07) | 0.04 (0.05) | 0.03 (0.03) | 0.02 (0.06) | 0.07 (0.11) | 0.02 (0.03) | 0.09 (0.06) | 0.10 (0.10) | 0.04 (0.06) | 0.03 (0.01) | 0.07 (0.06) | 0.04 (0.08) | 0.06 (0.07) | 0.04 (0.05) |
| fast | 1022 | 2.51 (1.53) | 2.33 (2.24) | 1.42 (1.28) | 1.44 (1.51) | 2.16 (1.27) | 2.32 (1.82) | 2.36 (1.18) | 2.34 (1.54) | 2.42 (1.78) | 2.13 (2.36) | 2.43 (1.11) | 2.70 (0.85) | 2.52 (1.53) | 2.33 (2.22) |
| Duration |  |  |  |  |  |  |  |  |  |  |  |  |  |  |  |
| all | 1025 | 0.89 (0.17) | 0.86 (0.18) | 0.79 (0.14) | 0.77 (0.29) | 0.99 (0.23) | 0.96 (0.24) | 0.98 (0.25) | 0.95 (0.38) | 0.88 (0.15) | 0.85 (0.08) | 0.85 (0.10) | 0.83 (0.12) | 0.89 (0.17) | 0.86 (0.18) |
| slow | 1024 | 0.99 (0.25) | 0.96 (0.27) | 0.95 (0.18) | 0.99 (0.35) | 1.00 (0.23) | 1.03 (0.06) | 0.99 (0.26) | 1.03 (0.34) | 1.00 (0.32) | 0.91 (0.30) | 0.92 (0.12) | 0.93 (0.19) | 0.99 (0.25) | 0.95 (0.27) |
| fast | 1022 | 0.89 (0.17) | 0.86 (0.18) | 0.79 (0.14) | 0.77 (0.29) | 0.98 (0.23) | 0.96 (0.23) | 0.97 (0.25) | 0.95 (0.38) | 0.88 (0.14) | 0.85 (0.08) | 0.84 (0.10) | 0.83 (0.13) | 0.89 (0.17) | 0.86 (0.18) |
| Frequency |  |  |  |  |  |  |  |  |  |  |  |  |  |  |  |
| all | 1025 | 13.77 (0.42) | 13.76 (0.58) | 13.88 (0.48) | 13.89 (0.88) | 13.86 (0.62) | 14.06 (0.90) | 13.43 (0.16) | 13.41 (0.24) | 14.01 (0.39) | 14.15 (0.39) | 13.67 (0.35) | 13.49 (0.51) | 13.77 (0.41) | 13.75 (0.57) |
| slow | 1024 | 11.78 (0.09) | 11.79 (0.09) | 11.80 (0.06) | 11.83 (0.12) | 11.74 (0.12) | 11.75 (0.16) | 11.80 (0.08) | 11.80 (0.12) | 11.79 (0.11) | 11.83 (0.17) | 11.79 (0.10) | 11.78 (0.16) | 11.78 (0.08) | 11.79 (0.09) |
| fast | 1022 | 13.82 (0.39) | 13.80 (0.55) | 13.90 (0.47) | 13.89 (0.82) | 13.90 (0.57) | 14.07 (0.88) | 13.50 (0.17) | 13.48 (0.27) | 14.03 (0.38) | 14.18 (0.39) | 13.72 (0.34) | 13.52 (0.49) | 13.81 (0.39) | 13.80 (0.54) |
| Amplitude |  |  |  |  |  |  |  |  |  |  |  |  |  |  |  |
| all | 1025 | 17.00 (35.9) | 10.17 (4.6) | 16.68 (19.9) | 9.09 (11.1) | 39.43 (82.9) | 10.52 (10.6) | 17.62 (11.0) | 13.65 (12.73) | 12.30 (8.4) | 10.83 (5.7) | 9.44 (1.8) | 9.75 (2.) | 16.87 (35.8) | 10.17 (4.5) |
| slow | 1024 | 19.68 (45.2) | 10.11 (5.3) | 23.88 (24.6) | 10.43 (43.4) | 15.10 (15.8) | 9.51 (5.5) | 14.77 (5.9) | 12.67 (6.8) | 15.16 (14.5) | 10.34 (7.8) | 9.46 (1.6) | 9.76 (2.) | 19.89 (46.1) | 10.10 (5.2) |
| fast | 1022 | 16.87 (35.6) | 10.15 (4.6) | 16.72 (20.0) | 9.10 (11.1) | 39.50 (82.9) | 10.52 (11.0) | 17.71 (11.1) | 13.71 (13.0) | 12.18 (8.0) | 10.82 (5.7) | 9.44 (1.9) | 9.75 (2.8) | 16.74 (35.3) | 10.14 (4.6) |
| **C4** |  |  |  |  |  |  |  |  |  |  |  |  |  |  |  |
| Density |  |  |  |  |  |  |  |  |  |  |  |  |  |  |  |
| all | 1020 | 2.23 (1.54) | 1.96 (2.03) | 0.93 (0.63) | 1.03 (1.00) | 1.75 (1.32) | 1.15 (1.70) | 2.39 (0.95) | 2.50 (1.54) | 3.15 (2.92) | 2.41 (1.57) | 2.16 (1.05) | 2.38 (1.08) | 2.23 (1.51) | 1.96 (2.06) |
| slow | 1020 | 0.06 (0.08) | 0.04 (0.05) | 0.02 (0.02) | 0.01 (0.02) | 0.07 (0.06) | 0.06 (0.07) | 0.07 (0.06) | 0.05 (0.06) | 0.06 (0.08) | 0.03 (0.05) | 0.10 (0.08) | 0.07 (0.06) | 0.06 (0.08) | 0.04 (0.05) |
| fast | 1018 | 2.18 (1.52) | 1.91 (1.97) | 0.92 (0.62) | 0.99 (1.01) | 1.71 (1.29) | 1.11 (1.61) | 2.32 (0.93) | 2.40 (1.48) | 3.10 (2.93) | 2.24 (1.56) | 2.06 (1.01) | 2.34 (1.03) | 2.18 (1.49) | 1.91 (2.02) |
| Duration |  |  |  |  |  |  |  |  |  |  |  |  |  |  |  |
| all | 1020 | 0.86 (0.17) | 0.83 (0.17) | 0.75 (0.12) | 0.71 (0.13) | 0.97 (0.27) | 0.91 (0.16) | 0.91 (0.17) | 0.85 (0.25) | 0.86 (0.12) | 0.86 (0.14) | 0.87 (0.11) | 0.86 (0.14) | 0.86 (0.16) | 0.83 (0.17) |
| slow | 1020 | 0.97 (0.25) | 0.93 (0.28) | 0.78 (0.14) | 0.77 (0.20) | 0.97 (0.32) | 0.91 (0.20) | 0.98 (0.10) | 0.99 (0.16) | 1.02 (0.23) | 1.04 (0.25) | 1.02 (0.15) | 1.04 (0.27) | 0.97 (0.26) | 0.93 (0.28) |
| fast | 1018 | 0.86 (0.16) | 0.83 (0.17) | 0.75 (0.12) | 0.71 (0.12) | 0.97 (0.27) | 0.91 (0.16) | 0.90 (0.18) | 0.85 (0.25) | 0.85 (0.12) | 0.87 (0.12) | 0.86 (0.12) | 0.85 (0.14) | 0.86 (0.16) | 0.82 (0.17) |
| Frequency |  |  |  |  |  |  |  |  |  |  |  |  |  |  |  |
| all | 1020 | 13.68 (0.40) | 13.65 (0.57) | 13.62 (0.34) | 13.53 (0.30) | 13.93 (0.58) | 13.90 (0.91) | 13.49 (0.32) | 13.40 (0.46) | 13.86 (0.47) | 13.96 (0.69) | 13.37 (0.22) | 13.30 (0.22) | 13.68 (0.40) | 13.65 (0.56) |
| slow | 1020 | 11.78 (0.08) | 11.79 (0.09) | 11.77 (0.12) | 11.74 (0.14) | 11.78 (0.08) | 11.77 (0.15) | 11.76 (0.04) | 11.76 (0.07) | 11.78 (0.08) | 11.79 (0.07) | 11.79 (0.05) | 11.78 (0.08) | 11.78 (0.08) | 11.79 (0.09) |
| fast | 1018 | 13.74 (0.38) | 13.70 (0.54) | 13.65 (0.34) | 13.53 (0.32) | 13.96 (0.55) | 13.93 (0.83) | 13.55 (0.31) | 13.46 (0.42) | 13.90 (0.45) | 14.04 (0.66) | 13.46 (0.21) | 13.46 (0.14) | 13.73 (0.37) | 13.71 (0.54) |
| Amplitude |  |  |  |  |  |  |  |  |  |  |  |  |  |  |  |
| all | 1020 | 18.00 (38.8) | 10.21 (4.7) | 16.85 (20.0) | 9.76 (10.4) | 19.86 (21.2) | 11.17 (9.2) | 17.91 (12.6) | 13.04 (13.9) | 13.00 (10.8) | 10.51 (5.1) | 9.40 (1.9) | 9.55 (2.8) | 18.15 (39.5) | 10.21 (4.7) |
| slow | 1020 | 20.48 (48.6) | 10.43 (5.4) | 20.54 (18.0) | 9.88 (15.7) | 19.33 (22.4) | 10.51 (9.3) | 18.15 (13.9) | 12.02 (15.2) | 14.83 (15.2) | 11.29 (8.5) | 9.98 (2.4) | 9.98 (3.8) | 20.69 (49.6) | 10.43 (5.7) |
| fast | 1018 | 17.69 (37.8) | 10.18 (4.6) | 16.86 (20.1) | 9.81 (10.1) | 19.86 (21.2) | 11.24 (9.3) | 17.89 (12.5) | 13.06 (13.9) | 12.86 (10.3) | 10.39 (5.1) | 9.37 (1.9) | 9.54 (2. 7) | 17.82 (38.6) | 10.18 (4.7) |

**Supplementary Table 4: Spearman's rho correlation coefficients of spindle parameters between electrodes**

|  | **ALL** | | | | | | | **SLOW** | | | | | | | **FAST** | | | | | | |
| --- | --- | --- | --- | --- | --- | --- | --- | --- | --- | --- | --- | --- | --- | --- | --- | --- | --- | --- | --- | --- | --- |
|  | **F3** | | **F4** | | **C3** | | **C4** | **F3** | | **F4** | | **C3** | | **C4** | **F3** | | **F4** | | **C3** | | **C4** |
|  | **rs** | ***p value*** | **rs** | ***p value*** | **rs** | ***p value*** | **rs** | **rs** | ***p value*** | **rs** | ***p value*** | **rs** | ***p value*** | **rs** | **rs** | ***p value*** | **rs** | ***p value*** | **rs** | ***p value*** | **rs** |
| **Density** |  |  |  |  |  |  |  |  |  |  |  |  |  |  |  |  |  |  |  |  |  |
| F3 | 1.0000 |  |  |  |  |  |  | 1.0000 |  |  |  |  |  |  | 1.0000 |  |  |  |  |  |  |
| F4 | 0.9108 | ***<0.001*** | 1.0000 |  |  |  |  | 0.8661 | ***<0.001*** | 1.0000 |  |  |  |  | 0.9130 | ***<0.001*** | 1.0000 |  |  |  |  |
| C3 | 0.7060 | ***<0.001*** | 0.6888 | ***<0.001*** | 1.0000 |  |  | 0.4772 | ***<0.001*** | 0.4595 | ***<0.001*** | 1.0000 |  |  | 0.7103 | ***<0.001*** | 0.6931 | ***<0.001*** | 1.0000 |  |  |
| C4 | 0.7237 | ***<0.001*** | 0.7458 | ***<0.001*** | 0.8324 | ***<0.001*** | 1.0000 | 0.5318 | ***<0.001*** | 0.5709 | ***<0.001*** | 0.5979 | ***<0.001*** | 1.0000 | 0.7258 | ***<0.001*** | 0.7500 | ***<0.001*** | 0.8320 | ***<0.001*** | 1.0000 |
| **Duration** |  |  |  |  |  |  |  |  |  |  |  |  |  |  |  |  |  |  |  |  |  |
| F3 | 1.0000 |  |  |  |  |  |  | 1.0000 |  |  |  |  |  |  | 1.0000 |  |  |  |  |  |  |
| F4 | 0.7295 | ***<0.001*** | 1.0000 |  |  |  |  | 0.5212 | ***<0.001*** | 1.0000 |  |  |  |  | 0.7267 | ***<0.001*** | 1.0000 |  |  |  |  |
| C3 | 0.4690 | ***<0.001*** | 0.4985 | ***<0.001*** | 1.0000 |  |  | 0.3308 | ***<0.001*** | 0.3492 | ***<0.001*** | 1.0000 |  |  | 0.4702 | ***<0.001*** | 0.5027 | ***<0.001*** | 1.0000 |  |  |
| C4 | 0.4960 | ***<0.001*** | 0.5463 | ***<0.001*** | 0.6434 | ***<0.001*** | 1.0000 | 0.3042 | ***<0.001*** | 0.3618 | ***<0.001*** | 0.3506 | ***<0.001*** | 1.0000 | 0.4956 | ***<0.001*** | 0.5507 | ***<0.001*** | 0.6507 | ***<0.001*** | 1.0000 |
| **Frequency** |  |  |  |  |  |  |  |  |  |  |  |  |  |  |  |  |  |  |  |  |  |
| F3 | 1.0000 |  |  |  |  |  |  | 1.0000 |  |  |  |  |  |  | 1.0000 |  |  |  |  |  |  |
| F4 | 0.9035 | ***<0.001*** | 1.0000 |  |  |  |  | 0.3089 | ***<0.001*** | 1.0000 |  |  |  |  | 0.8952 | ***<0.001*** | 1.0000 |  |  |  |  |
| C3 | 0.4476 | ***<0.001*** | 0.4251 | ***<0.001*** | 1.0000 |  |  | 0.0945 | ***<0.001*** | 0.1240 | ***<0.001*** | 1.0000 |  |  | 0.4228 | ***<0.001*** | 0.4018 | ***<0.001*** | 1.0000 |  |  |
| C4 | 0.4992 | ***<0.001*** | 0.5093 | ***<0.001*** | 0.7980 | ***<0.001*** | 1.0000 | 0.1318 | ***<0.001*** | 0.1792 | ***<0.001*** | 0.1233 | ***<0.001*** | 1.0000 | 0.4735 | ***<0.001*** | 0.4908 | ***<0.001*** | 0.7934 | ***<0.001*** | 1.0000 |
| **Max amplitude** |  |  |  |  |  |  |  |  |  |  |  |  |  |  |  |  |  |  |  |  |  |
| F3 | 1.0000 |  |  |  |  |  |  | 1.0000 |  |  |  |  |  |  | 1.0000 |  |  |  |  |  |  |
| F4 | 0.9447 | ***<0.001*** | 1.0000 |  |  |  |  | 0.8531 | ***<0.001*** | 1.0000 |  |  |  |  | 0.9445 | ***<0.001*** | 1.0000 |  |  |  |  |
| C3 | 0.8733 | ***<0.001*** | 0.8663 | ***<0.001*** | 1.0000 |  |  | 0.7853 | ***<0.001*** | 0.7483 | ***<0.001*** | 1.0000 |  |  | 0.8723 | ***<0.001*** | 0.8668 | ***<0.001*** | 1.0000 |  |  |
| C4 | 0.8442 | ***<0.001*** | 0.8635 | ***<0.001*** | 0.8854 | ***<0.001*** | 1.0000 | 0.7508 | ***<0.001*** | 0.7361 | ***<0.001*** | 0.7790 | ***<0.001*** | 1.0000 | 0.8460 | ***<0.001*** | 0.8649 | ***<0.001*** | 0.8886 | ***<0.001*** | 1.0000 |
